# 2,3-Dimethyl-*N*-[(*E*)-2,4,5-trimeth­oxy­benzyl­idene]aniline

**DOI:** 10.1107/S1600536810025894

**Published:** 2010-07-07

**Authors:** Abid Hussain, M. Nawaz Tahir, Muhammad Ilyas Tariq, Shahbaz Ahmad, Abdullah M. Asiri

**Affiliations:** a Department of Chemistry, Bahauddin Zakariya University, Multan 60800, Pakistan; b Department of Physics, University of Sargodha, Sargodha, Pakistan; c Department of Chemistry, University of Sargodha, Sargodha, Pakistan; d Chemistry Department, Faculty of Science, King Abdul Aziz University, PO Box 80203, Jeddah 21589, Saudi Arabia

## Abstract

In the title compound, C_18_H_21_NO_3_, the C=N bond has a *trans* conformation and the benzene rings are oriented at a dihedral angle of 61.32 (6)°. The C atoms of the three meth­oxy groups are all roughly coplanar with their attached ring [deviations = 0.219 (2), −0.097 (2) and −0.137 (2) Å]. In the crystal, a weak C—H⋯π inter­action may help to establish the packing.

## Related literature

For background information on Schiff bases and related crystal structures, see: Tahir *et al.* (2010*a* ▶,*b* ▶); Tariq *et al.* (2010 ▶).
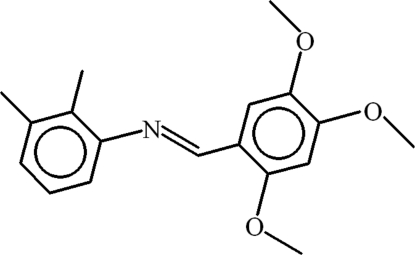


## Experimental

### 

#### Crystal data

C_18_H_21_NO_3_
*M* *_r_* = 299.36
Triclinic, 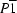

*a* = 7.0040 (2) Å
*b* = 11.0396 (4) Å
*c* = 11.1585 (4) Å
α = 73.941 (1)°
β = 76.022 (2)°
γ = 82.079 (1)°
*V* = 802.24 (5) Å^3^
*Z* = 2
Mo *K*α radiation
μ = 0.08 mm^−1^
*T* = 296 K
0.32 × 0.14 × 0.12 mm

#### Data collection

Bruker Kappa APEXII CCD diffractometer
Absorption correction: multi-scan (*SADABS*; Bruker, 2005 ▶) *T*_min_ = 0.980, *T*_max_ = 0.985
13855 measured reflections
3957 independent reflections
2935 reflections with *I* > 2σ(*I*)
*R*_int_ = 0.024

#### Refinement

*R*[*F* ^2^ > 2σ(*F* ^2^)] = 0.045
*wR*(*F* ^2^) = 0.139
*S* = 1.07
3957 reflections
204 parameters
H-atom parameters constrained
Δρ_max_ = 0.23 e Å^−3^
Δρ_min_ = −0.16 e Å^−3^

### 

Data collection: *APEX2* (Bruker, 2009 ▶); cell refinement: *SAINT* (Bruker, 2009 ▶); data reduction: *SAINT*; program(s) used to solve structure: *SHELXS97* (Sheldrick, 2008 ▶); program(s) used to refine structure: *SHELXL97* (Sheldrick, 2008 ▶); molecular graphics: *ORTEP-3* (Farrugia, 1997 ▶) and *PLATON* (Spek, 2009 ▶); software used to prepare material for publication: *WinGX* (Farrugia, 1999 ▶) and *PLATON*.

## Supplementary Material

Crystal structure: contains datablocks global, I. DOI: 10.1107/S1600536810025894/hb5535sup1.cif

Structure factors: contains datablocks I. DOI: 10.1107/S1600536810025894/hb5535Isup2.hkl

Additional supplementary materials:  crystallographic information; 3D view; checkCIF report

## Figures and Tables

**Table 1:** Hydrogen-bond geometry (Å, °) *Cg*1 is the centroid of the C1–C6 ring.

*D*—H⋯*A*	*D*—H	H⋯*A*	*D*⋯*A*	*D*—H⋯*A*
C16—H16*B*⋯*Cg*1^i^	0.96	2.99	3.5694 (19)	120

Symmetry code: (i) 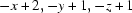.

**Fig. 1.**
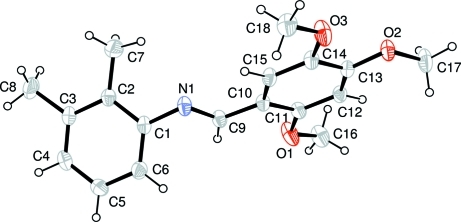
View of (I) with displacement ellipsoids drawn at the 30% probability level. H-atoms are shown by small circles of arbitrary radii.

## supplementary crystallographic information

### Comment

We have reported crystal structures of Schiff bases synthesized from 2,3-dimethylaniline (Tahir *et al.*, 2010*a*, 2010*b*), (Tariq *et al.*, 2010) and in continuation of this work, we report herein the structure and synthesis of the title compound (I, Fig. 1).

In (I) the 2,3-dimethylaniline moiety A (C1–C8/N1) and the group B (C9—C15/O1/O2/O3) of 2,4,5-trimethoxybenzaldehyde are planar with r. m. s. deviations of 0.0184 and 0.0103 Å, respectively. The dihedral angle between A/B is 61.32 (6)°. The title molecule essentially consists of monomers. The packing may be stabilized through weak C—H···π (Table 1) interactions.

### Experimental

Equimolar quantities of 2,3-dimethylaniline and 2,4,5-trimethoxybenzaldehyde were refluxed in methanol for 45 min resulting in violet solution. The solution was kept at room temperature which affoarded colorless prisms of (I) after 48 h.

### Refinement

The H-atoms were positioned geometrically (C–H = 0.93–0.96 Å) and refined as riding with *U*_iso_(H) = *xU*_eq_(C), where *x* = 1.5 for methyl and *x* = 1.2 for all other H-atoms.

### Crystal data

**Table:**

C_18_H_21_NO_3_	*Z* = 2
*M**_r_* = 299.36	*F*(000) = 320
Triclinic, *P*1	*D*_x_ = 1.239 Mg m^−^^3^
Hall symbol: -P 1	Mo *K*α radiation, λ = 0.71073 Å
*a* = 7.0040 (2) Å	Cell parameters from 2938 reflections
*b* = 11.0396 (4) Å	θ = 1.9–28.4°
*c* = 11.1585 (4) Å	µ = 0.08 mm^−^^1^
α = 73.941 (1)°	*T* = 296 K
β = 76.022 (2)°	Prism, colorless
γ = 82.079 (1)°	0.32 × 0.14 × 0.12 mm
*V* = 802.24 (5) Å^3^	

### Data collection

**Table:**

Bruker Kappa APEXII CCD diffractometer	3957 independent reflections
Radiation source: fine-focus sealed tube	2935 reflections with *I* > 2σ(*I*)
graphite	*R*_int_ = 0.024
Detector resolution: 7.5 pixels mm^-1^	θ_max_ = 28.4°, θ_min_ = 1.9°
ω scans	*h* = −7→9
Absorption correction: multi-scan (*SADABS*; Bruker, 2005)	*k* = −14→14
*T*_min_ = 0.980, *T*_max_ = 0.985	*l* = −14→14
13855 measured reflections	

### Refinement

**Table:**

Refinement on *F*^2^	Primary atom site location: structure-invariant direct methods
Least-squares matrix: full	Secondary atom site location: difference Fourier map
*R*[*F*^2^ > 2σ(*F*^2^)] = 0.045	Hydrogen site location: inferred from neighbouring sites
*wR*(*F*^2^) = 0.139	H-atom parameters constrained
*S* = 1.07	*w* = 1/[σ^2^(*F*_o_^2^) + (0.0685*P*)^2^ + 0.1056*P*] where *P* = (*F*_o_^2^ + 2*F*_c_^2^)/3
3957 reflections	(Δ/σ)_max_ < 0.001
204 parameters	Δρ_max_ = 0.23 e Å^−^^3^
0 restraints	Δρ_min_ = −0.16 e Å^−^^3^

### Special details

**Table:**

Geometry. Bond distances, angles *etc*. have been calculated using the rounded fractional coordinates. All su's are estimated from the variances of the (full) variance-covariance matrix. The cell e.s.d.'s are taken into account in the estimation of distances, angles and torsion angles
Refinement. Refinement of *F*^2^ against ALL reflections. The weighted *R*-factor *wR* and goodness of fit *S* are based on *F*^2^, conventional *R*-factors *R* are based on *F*, with *F* set to zero for negative *F*^2^. The threshold expression of *F*^2^ > σ(*F*^2^) is used only for calculating *R*-factors(gt) *etc*. and is not relevant to the choice of reflections for refinement. *R*-factors based on *F*^2^ are statistically about twice as large as those based on *F*, and *R*- factors based on ALL data will be even larger.

### Fractional atomic coordinates and isotropic or equivalent isotropic displacement parameters (Å^2^)

**Table:**

	*x*	*y*	*z*	*U*_iso_*/*U*_eq_	
O1	1.17927 (17)	0.38228 (10)	0.31146 (12)	0.0704 (4)	
O2	1.06960 (13)	0.10870 (9)	0.06502 (9)	0.0499 (3)	
O3	0.77474 (16)	0.25536 (10)	0.00283 (11)	0.0649 (4)	
N1	0.69274 (15)	0.60252 (10)	0.24254 (10)	0.0448 (3)	
C1	0.64619 (17)	0.71547 (11)	0.28410 (11)	0.0393 (3)	
C2	0.45691 (17)	0.73509 (12)	0.35802 (12)	0.0408 (4)	
C3	0.40689 (18)	0.84878 (12)	0.39403 (12)	0.0447 (4)	
C4	0.5412 (2)	0.94029 (13)	0.35392 (14)	0.0528 (4)	
C5	0.7257 (2)	0.92062 (13)	0.28035 (15)	0.0567 (5)	
C6	0.77862 (19)	0.80817 (13)	0.24555 (13)	0.0491 (4)	
C7	0.3148 (2)	0.63405 (16)	0.40020 (17)	0.0641 (6)	
C8	0.2084 (2)	0.87330 (18)	0.47689 (19)	0.0722 (6)	
C9	0.85705 (18)	0.54088 (11)	0.25246 (12)	0.0410 (4)	
C10	0.91943 (17)	0.42826 (11)	0.20390 (11)	0.0390 (3)	
C11	1.08153 (17)	0.34815 (11)	0.23517 (12)	0.0417 (4)	
C12	1.13627 (17)	0.23966 (11)	0.19044 (12)	0.0414 (3)	
C13	1.03063 (17)	0.21157 (11)	0.11341 (11)	0.0387 (3)	
C14	0.86709 (17)	0.29209 (12)	0.08024 (12)	0.0425 (4)	
C15	0.81463 (17)	0.39796 (12)	0.12541 (12)	0.0423 (4)	
C16	1.3221 (3)	0.29635 (15)	0.36533 (17)	0.0657 (6)	
C17	1.2405 (2)	0.02807 (15)	0.08431 (18)	0.0666 (6)	
C18	0.6215 (2)	0.33758 (15)	−0.04312 (16)	0.0591 (5)	
H4	0.50636	1.01641	0.37700	0.0633*	
H5	0.81429	0.98309	0.25425	0.0681*	
H6	0.90321	0.79454	0.19620	0.0589*	
H7A	0.30597	0.59426	0.48932	0.0961*	
H7B	0.36042	0.57209	0.35185	0.0961*	
H7C	0.18711	0.67105	0.38646	0.0961*	
H8A	0.20089	0.95488	0.49338	0.1082*	
H8B	0.19176	0.80943	0.55621	0.1082*	
H8C	0.10610	0.87119	0.43383	0.1082*	
H9	0.94031	0.56796	0.29169	0.0492*	
H12	1.24401	0.18624	0.21254	0.0496*	
H16	0.70660	0.45111	0.10338	0.0507*	
H16A	1.26434	0.21858	0.41271	0.0986*	
H16B	1.36937	0.33144	0.42145	0.0986*	
H16C	1.43011	0.28035	0.29862	0.0986*	
H17A	1.35460	0.07600	0.05100	0.1000*	
H17B	1.25245	−0.03752	0.04103	0.1000*	
H17C	1.23041	−0.00908	0.17400	0.1000*	
H18A	0.51745	0.34926	0.02752	0.0886*	
H18B	0.57116	0.30185	−0.09749	0.0886*	
H18C	0.67114	0.41771	−0.09075	0.0886*	

### Atomic displacement parameters (Å^2^)

**Table:**

	*U*^11^	*U*^22^	*U*^33^	*U*^12^	*U*^13^	*U*^23^
O1	0.0813 (7)	0.0513 (6)	0.1068 (9)	0.0226 (5)	−0.0673 (7)	−0.0385 (6)
O2	0.0521 (5)	0.0475 (5)	0.0571 (6)	0.0132 (4)	−0.0173 (4)	−0.0280 (4)
O3	0.0709 (6)	0.0633 (6)	0.0850 (8)	0.0239 (5)	−0.0489 (6)	−0.0447 (6)
N1	0.0471 (5)	0.0455 (6)	0.0486 (6)	0.0100 (4)	−0.0174 (5)	−0.0234 (5)
C1	0.0439 (6)	0.0405 (6)	0.0376 (6)	0.0082 (5)	−0.0159 (5)	−0.0157 (5)
C2	0.0414 (6)	0.0433 (7)	0.0412 (6)	0.0045 (5)	−0.0152 (5)	−0.0145 (5)
C3	0.0444 (6)	0.0457 (7)	0.0455 (7)	0.0107 (5)	−0.0129 (5)	−0.0179 (5)
C4	0.0636 (8)	0.0377 (7)	0.0588 (8)	0.0078 (6)	−0.0145 (7)	−0.0195 (6)
C5	0.0616 (8)	0.0425 (7)	0.0634 (9)	−0.0089 (6)	−0.0045 (7)	−0.0143 (6)
C6	0.0477 (7)	0.0493 (8)	0.0471 (7)	0.0008 (6)	−0.0032 (5)	−0.0151 (6)
C7	0.0542 (8)	0.0645 (10)	0.0787 (11)	−0.0084 (7)	−0.0076 (7)	−0.0305 (8)
C8	0.0537 (8)	0.0756 (11)	0.0892 (12)	0.0108 (8)	−0.0016 (8)	−0.0435 (10)
C9	0.0447 (6)	0.0393 (6)	0.0437 (7)	0.0041 (5)	−0.0166 (5)	−0.0157 (5)
C10	0.0398 (6)	0.0378 (6)	0.0417 (6)	0.0044 (5)	−0.0124 (5)	−0.0141 (5)
C11	0.0427 (6)	0.0398 (6)	0.0483 (7)	0.0020 (5)	−0.0193 (5)	−0.0145 (5)
C12	0.0366 (5)	0.0388 (6)	0.0498 (7)	0.0066 (4)	−0.0140 (5)	−0.0133 (5)
C13	0.0387 (5)	0.0383 (6)	0.0392 (6)	0.0039 (4)	−0.0063 (5)	−0.0150 (5)
C14	0.0420 (6)	0.0463 (7)	0.0449 (7)	0.0059 (5)	−0.0164 (5)	−0.0192 (5)
C15	0.0388 (6)	0.0444 (7)	0.0477 (7)	0.0100 (5)	−0.0164 (5)	−0.0185 (5)
C16	0.0718 (9)	0.0584 (9)	0.0833 (11)	0.0144 (7)	−0.0510 (9)	−0.0240 (8)
C17	0.0627 (9)	0.0577 (9)	0.0902 (12)	0.0253 (7)	−0.0277 (8)	−0.0407 (9)
C18	0.0555 (8)	0.0674 (9)	0.0649 (9)	0.0080 (7)	−0.0316 (7)	−0.0240 (8)

### Geometric parameters (Å, °)

**Table:**

O1—C11	1.3658 (18)	C14—C15	1.3686 (19)
O1—C16	1.406 (2)	C4—H4	0.9300
O2—C13	1.3559 (16)	C5—H5	0.9300
O2—C17	1.4129 (19)	C6—H6	0.9300
O3—C14	1.3656 (17)	C7—H7A	0.9600
O3—C18	1.407 (2)	C7—H7B	0.9600
N1—C1	1.4181 (17)	C7—H7C	0.9600
N1—C9	1.2664 (17)	C8—H8A	0.9600
C1—C2	1.4058 (17)	C8—H8B	0.9600
C1—C6	1.3849 (19)	C8—H8C	0.9600
C2—C3	1.3962 (19)	C9—H9	0.9300
C2—C7	1.499 (2)	C12—H12	0.9300
C3—C4	1.384 (2)	C15—H16	0.9300
C3—C8	1.508 (2)	C16—H16A	0.9600
C4—C5	1.378 (2)	C16—H16B	0.9600
C5—C6	1.378 (2)	C16—H16C	0.9600
C9—C10	1.4625 (18)	C17—H17A	0.9600
C10—C11	1.3917 (18)	C17—H17B	0.9600
C10—C15	1.3993 (18)	C17—H17C	0.9600
C11—C12	1.3937 (18)	C18—H18A	0.9600
C12—C13	1.3785 (17)	C18—H18B	0.9600
C13—C14	1.4076 (18)	C18—H18C	0.9600
			
C11—O1—C16	119.52 (12)	C2—C7—H7A	109.00
C13—O2—C17	118.84 (11)	C2—C7—H7B	109.00
C14—O3—C18	117.70 (12)	C2—C7—H7C	109.00
C1—N1—C9	119.09 (11)	H7A—C7—H7B	109.00
N1—C1—C2	118.33 (11)	H7A—C7—H7C	109.00
N1—C1—C6	120.89 (11)	H7B—C7—H7C	109.00
C2—C1—C6	120.63 (12)	C3—C8—H8A	109.00
C1—C2—C3	118.61 (12)	C3—C8—H8B	109.00
C1—C2—C7	120.36 (12)	C3—C8—H8C	109.00
C3—C2—C7	121.00 (12)	H8A—C8—H8B	109.00
C2—C3—C4	119.75 (12)	H8A—C8—H8C	109.00
C2—C3—C8	120.94 (13)	H8B—C8—H8C	109.00
C4—C3—C8	119.31 (13)	N1—C9—H9	119.00
C3—C4—C5	121.16 (13)	C10—C9—H9	119.00
C4—C5—C6	119.86 (14)	C11—C12—H12	120.00
C1—C6—C5	119.98 (13)	C13—C12—H12	120.00
N1—C9—C10	121.79 (12)	C10—C15—H16	119.00
C9—C10—C11	121.37 (11)	C14—C15—H16	119.00
C9—C10—C15	120.23 (11)	O1—C16—H16A	109.00
C11—C10—C15	118.40 (11)	O1—C16—H16B	109.00
O1—C11—C10	116.09 (11)	O1—C16—H16C	109.00
O1—C11—C12	123.28 (12)	H16A—C16—H16B	109.00
C10—C11—C12	120.63 (11)	H16A—C16—H16C	109.00
C11—C12—C13	119.97 (12)	H16B—C16—H16C	109.00
O2—C13—C12	125.10 (11)	O2—C17—H17A	109.00
O2—C13—C14	114.82 (11)	O2—C17—H17B	109.00
C12—C13—C14	120.08 (12)	O2—C17—H17C	109.00
O3—C14—C13	114.95 (12)	H17A—C17—H17B	109.00
O3—C14—C15	125.80 (12)	H17A—C17—H17C	110.00
C13—C14—C15	119.25 (12)	H17B—C17—H17C	109.00
C10—C15—C14	121.67 (12)	O3—C18—H18A	109.00
C3—C4—H4	119.00	O3—C18—H18B	109.00
C5—C4—H4	119.00	O3—C18—H18C	110.00
C4—C5—H5	120.00	H18A—C18—H18B	109.00
C6—C5—H5	120.00	H18A—C18—H18C	109.00
C1—C6—H6	120.00	H18B—C18—H18C	109.00
C5—C6—H6	120.00		
			
C16—O1—C11—C10	169.05 (13)	C3—C4—C5—C6	0.1 (2)
C16—O1—C11—C12	−10.5 (2)	C4—C5—C6—C1	0.3 (2)
C17—O2—C13—C12	−5.76 (19)	N1—C9—C10—C11	−168.10 (12)
C17—O2—C13—C14	174.79 (12)	N1—C9—C10—C15	11.16 (19)
C18—O3—C14—C13	−174.38 (12)	C9—C10—C11—O1	−1.04 (18)
C18—O3—C14—C15	5.2 (2)	C9—C10—C11—C12	178.53 (12)
C9—N1—C1—C2	−134.93 (13)	C15—C10—C11—O1	179.69 (12)
C9—N1—C1—C6	49.51 (17)	C15—C10—C11—C12	−0.75 (18)
C1—N1—C9—C10	−175.98 (11)	C9—C10—C15—C14	−178.84 (12)
N1—C1—C2—C3	−176.94 (11)	C11—C10—C15—C14	0.45 (19)
N1—C1—C2—C7	4.86 (18)	O1—C11—C12—C13	−179.82 (12)
C6—C1—C2—C3	−1.38 (18)	C10—C11—C12—C13	0.65 (19)
C6—C1—C2—C7	−179.57 (13)	C11—C12—C13—O2	−179.65 (12)
N1—C1—C6—C5	175.87 (12)	C11—C12—C13—C14	−0.23 (18)
C2—C1—C6—C5	0.4 (2)	O2—C13—C14—O3	−0.99 (16)
C1—C2—C3—C4	1.67 (19)	O2—C13—C14—C15	179.40 (11)
C1—C2—C3—C8	−177.99 (13)	C12—C13—C14—O3	179.53 (11)
C7—C2—C3—C4	179.86 (13)	C12—C13—C14—C15	−0.07 (19)
C7—C2—C3—C8	0.2 (2)	O3—C14—C15—C10	−179.60 (12)
C2—C3—C4—C5	−1.0 (2)	C13—C14—C15—C10	0.0 (2)
C8—C3—C4—C5	178.63 (14)		

### Hydrogen-bond geometry (Å, °)

**Table:**

*D*—H···*A*	*D*—H	H···*A*	*D*···*A*	*D*—H···*A*
C16—H16B···Cg1^i^	0.96	2.99	3.5694 (19)	120

Symmetry codes: (i) −*x*+2, −*y*+1, −*z*+1.
